# Genotypic Resistance Analysis of Bacterial Species Involved in Infectious Keratitis

**DOI:** 10.3390/diagnostics16010135

**Published:** 2026-01-01

**Authors:** Andrei Theodor Bălășoiu, Ovidiu Mircea Zlatian, Maria Balasoiu, Andrei Osman, Alice Elena Ghenea, Ramona Cioboată, Simona-Daniela Neamtu, Andreea Loredana Golli

**Affiliations:** 1 Ophthalmology Department, County Clinical Emergency Hospital of Craiova, 200642 Craiova, Romania; andrei_theo@yahoo.com; 2 Ophthalmology Department, University of Medicine and Pharmacy of Craiova, 200349 Craiova, Romania; 3 Medical Laboratory, County Clinical Emergency Hospital of Craiova, 200642 Craiova, Romania; maria.balasoiu@umfcv.ro (M.B.); gaman_alice@yahoo.com (A.E.G.); 4 Microbiology Department, University of Medicine and Pharmacy of Craiova, 200349 Craiova, Romania; 5 ENT Clinic, County Clinical Emergency Hospital of Craiova, 200642 Craiova, Romania; andrei.osman@umfcv.ro; 6 ENT Department, University of Medicine and Pharmacy of Craiova, 200349 Craiova, Romania; 7 Department of Pneumology, University of Medicine and Pharmacy of Craiova, 200349 Craiova, Romania; ramona_cioboata@yahoo.com; 8 Department of Pneumology, Victor Babes University Hospital, 200515 Craiova, Romania; 9 Department Immunology, Hematology, Faculty of Pharmacy, University of Medicine and Pharmacy of Craiova, 200638 Craiova, Romania; simona.neamtu@umfcv.ro; 10 Medical Laboratory, Clinical Municipal Hospital “Filantropia” of Craiova, 200143 Craiova, Romania; 11 Department for Prevention of Infections Associated with Healthcare, County Clinical Emergency Hospital of Craiova, 200642 Craiova, Romania; andreea_golli@yahoo.com; 12 Public Health Department, University of Medicine and Pharmacy of Craiova, 200349 Craiova, Romania

**Keywords:** infectious keratitis, bacterial pathogens, antimicrobial resistance, resistance genes, ocular microbiology

## Abstract

**Background/Objectives**: Infectious keratitis represents a major ophthalmological emergency and a leading cause of corneal blindness worldwide. Rapid progression, frequent antimicrobial resistance, and poor therapeutic outcomes make genotypic surveillance essential. This study aimed to analyze the distribution of bacterial pathogens involved in infectious keratitis and characterize their resistance mechanisms at the genotypic level, with emphasis on extended-spectrum β-lactamase (ESBL) genes. **Methods**: Corneal scrapings were collected from patients diagnosed with bacterial keratitis at the County Clinical Emergency Hospital of Craiova. Isolates were identified using standard microbiological techniques, followed by antimicrobial susceptibility testing. Genotypic resistance markers, including tem, shv, and *ctx-M* gene families, were investigated using PCR-based methods, while phenotypic resistance was investigated using the Vitek2 system. **Results**: Gram-positive bacteria were the predominant isolates, with coagulase-negative staphylococci and *Staphylococcus aureus* accounting for most cases. Among Gram-negative pathogens, *Pseudomonas aeruginosa* and *Enterobacteriaceae* were frequently detected. Genotypic analysis revealed tem genes in a substantial proportion of ocular isolates, often plasmid-mediated, while *shv* genes showed low prevalence and *ctx-M* genes were less frequent. The genetic determinants correlated well with phenotypic resistance expressed by MICs. **Conclusions**: The study highlights the significant correlation between genotypic markers and resistance phenotypes in bacterial keratitis. Continuous molecular surveillance is essential to guide targeted therapy, prevent therapeutic failure, and improve patient outcomes in infectious keratitis.

## 1. Introduction

Bacterial keratitis is one of the most severe ophthalmological emergencies, constituting a major cause of ocular morbidity and irreversible vision loss globally. Corneal opacity, largely resulting from corneal infections, is recognized as the fifth-leading cause of blindness and visual impairment worldwide, affecting approximately six million people globally. Annually, between 1.5 and 2.0 million new cases of monocular blindness are reported, highlighting a continuous and unmitigated burden on human health. Among all etiologies that can affect the cornea, including infections, trauma, inflammation, degeneration, and nutritional deficiencies, infectious keratitis is the primary cause of corneal blindness in both developed and developing countries [1,2,3].

The incidence of bacterial keratitis shows significant geographical variations, reflecting differences in socio-economic conditions, access to healthcare services, and region-specific risk factors. In developed countries, the estimated incidence ranges from 2.5 to 40.3 cases per 100,000 of the population per year. In contrast, developing countries report substantially higher incidences, with values of 113 per 100,000 per year in South India and up to 799 per 100,000 per year in Nepal [1,4,5,6,7].

The etiological spectrum of bacterial keratitis is dominated by Gram-positive cocci, particularly *Staphylococcus aureus* and coagulase-negative staphylococci, which are the most frequently isolated pathogens in most epidemiological studies. This predominance of Gram-positive cocci is consistently observed in various geographical and clinical contexts, although the specific distribution may vary depending on local risk factors and the characteristics of the studied population. Alongside these main agents, Gram-negative bacilli, including *Pseudomonas aeruginosa*, *Klebsiella* spp., and other enterobacteria, constitute a significant proportion of bacterial isolates, often associated with specific risk factors such as contact lens wear or ocular trauma [8,9,10].

The risk factors for developing bacterial keratitis are multiple and interconnected, reflecting the complexity of the pathogenesis of these infections: contact lens wear, ocular trauma, prior ocular surgery, ocular surface diseases (including dry eye), age, systemic comorbidities (e.g., diabetes), or immunosuppression [11].

Antimicrobial resistance (AMR) is an intrinsic or acquired characteristic, encoded by genes that can be transferred between bacteria, constituting a global phenomenon that has made some systemic bacterial infections difficult to treat. The emergence of antibiotic resistance in bacterial keratitis represents one of the greatest contemporary challenges in the management of these infections, significantly limiting therapeutic options and compromising the visual prognosis of patients. In the ophthalmological context, an alarming increase in the prevalence of bacterial resistance to fluoroquinolones, beta-lactams, and aminoglycosides has been observed in ophthalmic isolates [10,12,13,14].

In this complex and continuously evolving context, the analysis of the incidence, etiology, and antibiotic resistance of bacterial keratitis becomes essential for guiding empirical therapy and improving the visual prognosis of patients. Knowledge of the local resistance profile is crucial for optimizing therapeutic strategies and preventing treatment failures that can lead to severe complications, including corneal perforation, endophthalmitis, and loss of the eyeball. Knowledge of the specific etiological agent can guide clinical suspicion and inform therapeutic decisions in the absence of an immediate microbiological result.

Although AMR trends in ocular isolates are periodically reported, most work focuses on resistance phenotypes, with far fewer studies profiling resistance genes on the ocular surface or in keratitis. Recent reviews explicitly note that ocular microbiome/AMR knowledge remains relatively early-stage compared with other body sites [13].

In this context, there is a clear unmet need for studies that integrate molecular identification of bacterial pathogens and resistance determinants directly from ocular samples. Conventional culture-based diagnostics, while essential, are time-consuming and may fail to detect fastidious or non-culturable organisms, leading to delayed or suboptimal treatment decisions. By incorporating multiplex polymerase chain reaction (PCR)-based molecular assays it becomes possible to rapidly detect both bacterial species and key resistance genes within hours, providing actionable data before classical phenotypic results are available.

This study aims to investigate the incidence, etiological agents, and resistance gene profiles associated with bacterial keratitis, and to correlate these findings with phenotypic resistance patterns. The overarching objective is to generate clinically relevant insights that support the rapid diagnosis of bacterial keratitis and the identification of antimicrobial resistance, thereby enabling prompt and tailored therapeutic interventions. The results obtained will inform the development of locally adapted clinical practice guidelines and contribute to optimizing prevention and treatment strategies for bacterial keratitis.

## 2. Materials and Methods

### 2.1. Study Design

This study represents a retrospective observational study, conducted to evaluate the incidence, etiology, and antibiotic resistance profile of bacterial keratitis. The study can be particularly valuable in the context of epidemiological surveillance, allowing the identification of temporal trends and the resistance genes of bacteria that cause corneal infections.

The study included a total of 176 eyes from 176 patients who were hospitalized between 1 July 2024 and 30 June 2025 in the Ophthalmology Clinic of the Clinical Emergency Hospital of Craiova, Romania. Patient selection was based on the presence of clinical signs and symptoms compatible with bacterial keratitis, including conjunctival hyperemia, purulent discharge, ocular pain, photophobia, decreased visual acuity, and suggestive corneal clinical aspect at slit lamp examination.

Inclusion criteria: The presence of clinical signs of corneal infection, patient consent for the collection of biological samples, availability of complete clinical data, and the possibility of clinical follow-up.

Exclusion criteria: Patients with a history of allergy to dyes used in microscopic examinations, and patients for whom sample collection could not be performed for technical reasons were excluded from the study.

The collection of clinical and demographic data was conducted through a systematic review of the medical records of patients included in the study.

### 2.2. Sample Collection

Corneal scraping collection was performed in the Ophthalmology Clinic via slit lamp and under topical anesthesia, with the help of a 20G needle. Samples were collected before the initiation of any topical or systemic antimicrobial treatment to avoid interference with bacterial growth and to ensure the maximum sensitivity of detection methods. The collection was performed with strict adherence to aseptic and antiseptic measures to prevent sample contamination.

Transport of samples to the microbiology laboratory was carried out as quickly as possible to maintain the viability of microorganisms and prevent the proliferation of contaminants.

The samples were first inoculated on appropriate culture media for microbiological diagnosis and then subjected to a rapid molecular assay for identification of bacteria and antibiotic resistance genes. The molecular assay identified bacteria only at genus level, the definitive identification was performed on the biochemical identification system.

### 2.3. Molecular Identification of Bacteria and Resistance Genes

Corneal scrapings were analyzed using the Unyvero platform (Curetis/OpGen). Briefly, samples were suspended in the Unyvero sample tube, lysed (Unyvero L4 Lysator, ~30 min), and loaded into an application-specific cartridge run on an A50 Analyzer (Curetis, Holzgerlingen, Germany), with results visualized in C8 Cockpit software, version 6.0; the assay used high-multiplex PCR followed by array hybridization. The Unyvero system identifies genetic target sequences linked to specific bacterial species and molecular antibiotic resistance markers. The whole procedure took up to 5 h. The cartridge panels used in this study target a broad spectrum of Gram-positive and Gram-negative bacteria relevant to ocular infections and report AMR markers including *mecA*/*mecC* (methicillin resistance), *vanA*/*vanB* (glycopeptide resistance), ESBLs (*tem*, *shv*, *ctx-M*), aminoglycosides resistance (*aac2*, *aacAc4*), plasmid-mediated quinolone resistance (*qnrA*/*B*/*S*, *gyrA83*/*87*), macrolide resistance (*ermA*, *ermC*), sulfonamides resistance (sul1), and carbapenemases (*kpc*, *ndm*, *vim*, *oxa-23*, *oxa-24*/*40* and *oxa-48*) [15].

### 2.4. Bacterial Culture and Identification

Inoculation of samples was performed on standard and selective culture media, adapted for the optimal growth of a wide range of pathogenic bacteria. The culture media used included blood agar, which served as a base medium for the growth of most bacteria and for highlighting hemolytic properties, and chocolate agar, optimal for the growth of fastidious bacteria. Selective media, such as MacConkey agar for the isolation and differentiation of enterobacteria and non-glucose-fermenters, were also used.

Incubation of cultures was performed under standard temperature (37 °C) and atmospheric (aerobic and microaerophilic) conditions, with daily monitoring of bacterial growth for up to 5–7 days, before being reported as negative, to allow the growth of slow-developing bacteria.

Preliminary identification of bacteria was based on the morphological characteristics of colonies (size, shape, color, surface appearance, and hemolytic properties), microscopic examination of Gram-stained smears made from pure colonies, and rapid biochemical tests. Confirmation of identification was achieved by automated phenotypic profiling using the VITEK 2 system (Biomerieux, Marcy-l’Étoile, France), which relies on miniaturized biochemical reagent cards (identification cards: GN, GP, ANC, and NH; antibiotic susceptibility testing cards: for Gram positives AST-P592, and AST-N438, AST-XN26 for Gram negatives) inoculated with standardized microbial suspensions at 0.5 McFarlands. Therapeutic interpretation of MICs was performed using EUCAST guidelines.

**Figure 1:**
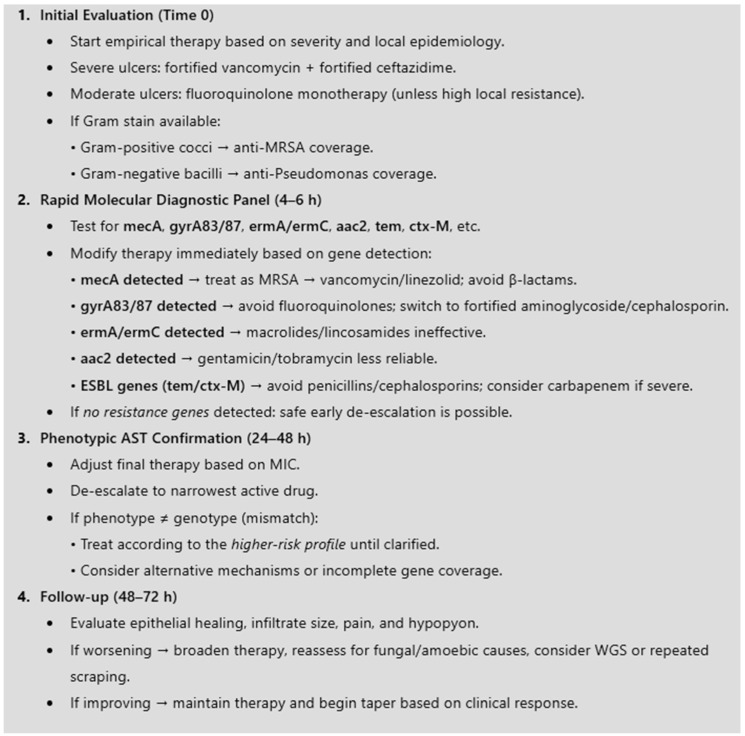
Evidence-based treatment algorithm for bacterial keratitis integrating rapid molecular diagnostics. AST—Antibiotic Susceptibility Testing; ESBL—Extended Spectrum Beta Lactamases; MIC—Minimum inhibitory concentration; MRSA—Methicillin-resistant *Staphylococcus aureus*; WGS—Whole-Genome Sequencing.

The evidence-based management algorithm used for bacterial keratitis integrated rapid molecular diagnostics and is presented in Figure 1.

### 2.5. Statistical Analysis

The statistical analysis of the data was performed using specialized software for statistical analysis, applying appropriate descriptive and inferential methods for the type and distribution of the collected data. The objective of the statistical analysis was to characterize antibiotic resistance profiles by correlating genotypic and phenotypic resistance.

Descriptive statistics included the calculation of absolute and relative frequencies for categorical variables, as well as measures of central tendency and dispersion for continuous variables. Analysis of gene co-occurrence used the Jaccard index which quantifies the similarity between gene sets by calculating the size of their intersection divided by the size of their union.

Odds ratios (OR), 95% confidence intervals (CI), and *p*-values were calculated for each gene–antibiotic pair, both by Fischer’s test. CMI distribution was analyzed by logistic regression.

The level of statistical significance was set at *p* < 0.05 for all tests performed. An analysis of missing data was conducted to evaluate their impact on the validity of the results and to apply appropriate methods for handling incomplete data.

### 2.6. Ethical Considerations

The study was conducted in accordance with the fundamental ethical principles for medical research, respecting the Declaration of Helsinki and local regulations regarding clinical research. The study was approved by the Ethical Committee of the Clinical Emergency Hospital of Craiova, Romania, and the University of Medicine and Pharmacy of Craiova, no. 38/3 September 2025. As an observational study that did not involve experimental interventions or modifications to standard clinical management, the risks to patients were minimal.

Patient data confidentiality was ensured by anonymizing personal information and using identification codes for statistical analysis. Access to clinical data was restricted to authorized research personnel, strictly adhering to data protection regulations.

Informed consent for study participation was obtained in accordance with the institution’s standard procedures, and as the study site is a teaching hospital, each patient was informed of data usage methods. Study data will be securely stored in accordance with current regulations and will be available for further analysis in ethically approved research projects.

## 3. Results

Of the 176 corneal samples collected, we identified bacterial pathogens in 136 (77.27%), while the other 40 samples (22.73%) were negative.

### 3.1. Etiological Spectrum of Bacterial Keratitis

**Figure 2:**
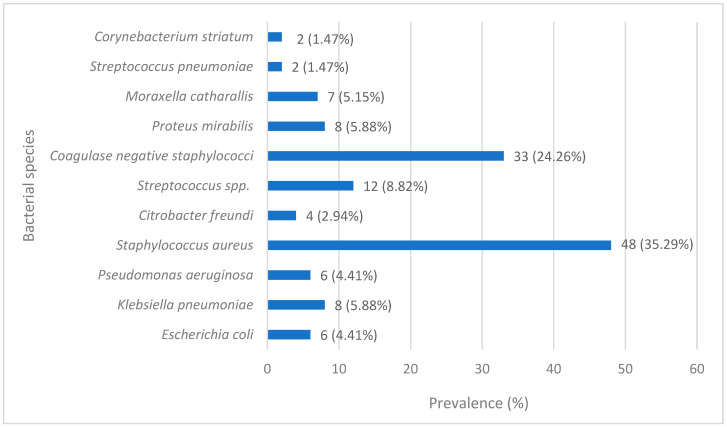
Percentage distribution of bacterial species isolated from corneal scrapings. The clear predominance of Gram-positive cocci, especially *Staphylococcus aureus*, compared to Gram-negative bacilli, is highlighted.

The etiological spectrum (Figure 2) shows the predominance of Gram-positive cocci (69.85%), represented by *Staphylococcus aureus* (48 isolates), coagulase-negative staphylococci (33 isolates) and *Streptococcus* spp. (12 isolates), underscoring their major role as causative agents of bacterial keratitis. Among Gram-negative organisms, *Klebsiella pneumoniae* (8 isolates) were the most frequently identified, followed by *Proteus mirabilis* (8 isolates), *Moraxella catarrhalis* (7 isolates), and *Pseudomonas aeruginosa* (6 isolates).

Less common isolates, including *Escherichia coli*, *Citrobacter freundii*, and *Corynebacterium striatum*, reflect the wide microbiological diversity of bacterial keratitis and suggest that local epidemiology may be influenced by environmental exposure, host factors, and healthcare practices.

### 3.2. Molecular Analysis

**Figure 3:**
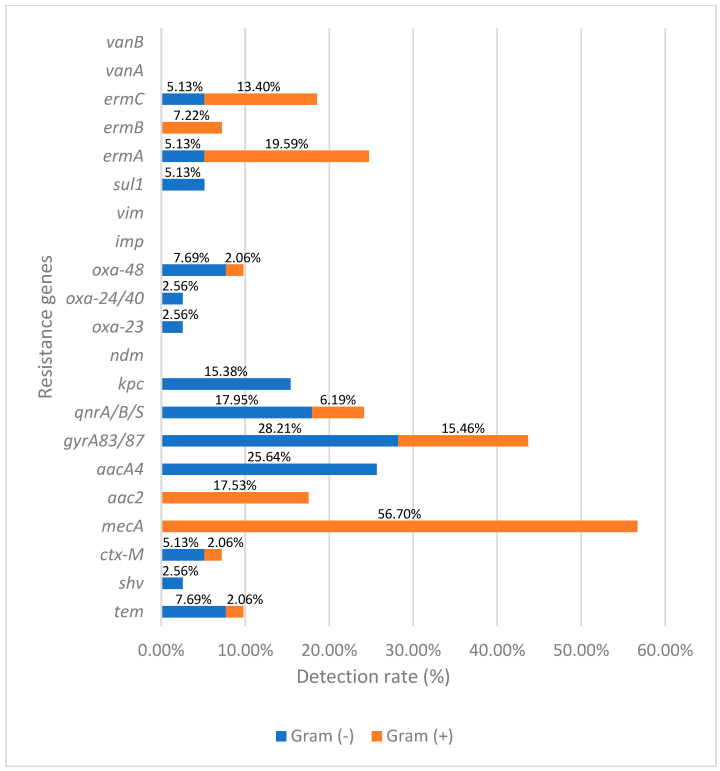
Antibiotic resistance genes identified in clinical isolates.

The molecular analysis identified 21 distinct resistance genes across the isolates (Figure 3, Table 1). The most prevalent was *mecA*, detected in 55 isolates (over 60% of the total), mainly in *Staphylococcus aureus* (34 isolates) and coagulase-negative staphylococci (21 isolates), confirming the high burden of methicillin resistance among staphylococcal keratitis cases. Genes mediating macrolide resistance were also frequent: *ermA* (21 isolates), *ermC* (15 isolates), and *ermB* (7 isolates), reflecting a substantial prevalence of resistance to erythromycin and related agents. Fluoroquinolone resistance genes were widely distributed, especially *gyrA83*/*87* (27 isolates) and *qnrA*/*B*/*S* (19 isolates), most notably in *E. coli* and *K. pneumoniae* (4 and 6 isolates, respectively), *Moraxella catarrhalis* (3 isolates), and coagulase negative staphylococci (5 isolates).

Aminoglycoside resistance was less common but still significant, with the aac2 gene (17 isolates) and *aacA4* gene (9 isolates) predominantly found in coagulase-negative staphylococci. Extended-spectrum β-lactamase genes were rare, with tem (5 isolates), *ctx-M* (4 isolates), and *shv* (1 isolate) detected mainly in Gram-negative bacilli such as *K. pneumoniae* and *E. coli*. None of the *E. coli* isolates harbored the *sul1* gene, which is responsible for sulfonamide resistance.

Carbapenemase genes were uncommon: *oxa-48* was found in 5 isolates, *oxa-23* in 1 isolate, *kpc* in one isolate, while *ndm*, *imp*, and *vim* were absent. The relatively low prevalence of carbapenemase genes suggests that carbapenem resistance has not yet become widespread in keratitis pathogens, though the sporadic detection of oxa-48 emphasizes the need for ongoing surveillance.

Even though *erm* genes, which confer resistance to macrolides, were detected in four *Moraxella* strains, these antibiotics were not tested phenotypically because they were not included on AST cards for Gram negatives. One of the strains was resistant to sulphamethoxazole-thrimethoprim even though gene *sul1* was not detected.

*Corynebacterium striatum* was identified by the Vitek2 system but antibiotic resistance was not tested.

### 3.3. Gene–Gene Correlation and Co-Occurrence

**Figure 4:**
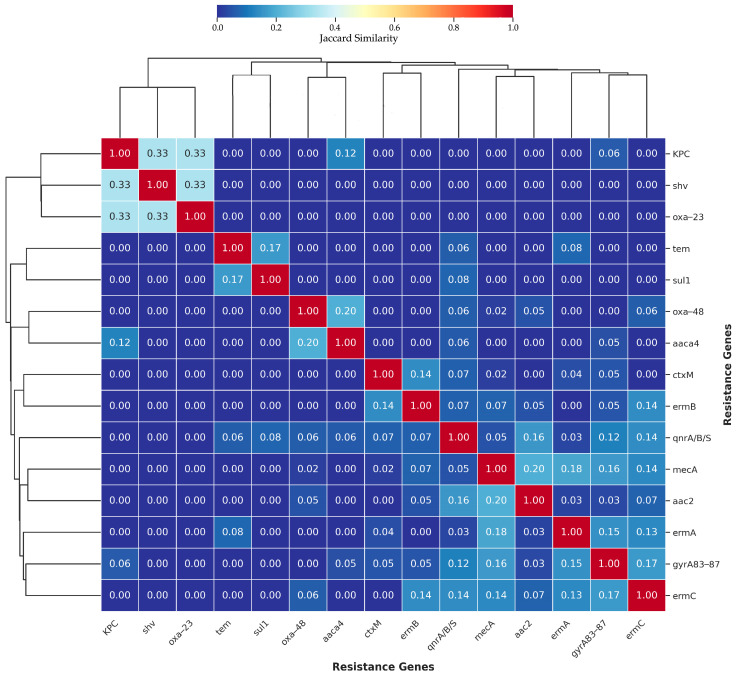
Heatmap of gene co-occurrence patterns (Jaccard Index). Higher values indicate more frequent co-occurrence.

Hierarchical clustering analysis based on Jaccard similarity (Figure 4) revealed distinct patterns of antimicrobial resistance gene associations among the bacterial isolates. The analysis identified both clustered resistance genes and independently occurring genes, suggesting co-selection and indicating diverse resistance mechanisms across the study population. The analysis revealed a notable cluster comprising *kpc*, *shv*, and *oxa-23* genes, which exhibited moderate similarity indices (Jaccard similarity = 0.33). This co-occurrence pattern suggests these carbapenemase and extended-spectrum beta-lactamase genes may be harbored on shared mobile genetic elements or selected together under carbapenem pressure. In contrast, other beta-lactamase genes showed more isolated distributions, with *tem* and *sul1* genes displaying weak association (Jaccard similarity = 0.17), while *oxa-48* demonstrated limited co-occurrence with *aac4* (Jaccard similarity = 0.20).

A distinct cluster emerged comprising quinolone resistance genes (*qnrA*/*B*/*S*), methicillin resistance (*mecA*), and aminoglycoside resistance genes (*aac2*), with Jaccard similarity values ranging from 0.16 to 0.20. This pattern indicates these resistance determinants frequently co-exist in the same bacterial strains, suggesting the presence of multidrug-resistant isolates carrying resistance to multiple antibiotic classes. The *ctxM* and ermB genes also showed co-occurrence (Jaccard similarity = 0.14), indicating simultaneous carriage of extended-spectrum beta-lactamase and macrolide resistance mechanisms.

The *ermA*, gyrA83_87, and *ermC* genes formed a loosely associated cluster, with Jaccard similarity values between 0.13 and 0.17, suggesting moderate co-occurrence of macrolide resistance genes with fluoroquinolone resistance mutations. This pattern may reflect selection pressure from the clinical use of both antibiotic classes or their co-localization on mobile genetic elements.

The majority of gene pairs demonstrated very low Jaccard similarity values (0.00), indicating independent occurrence across the bacterial population. This finding suggests that while certain resistance genes cluster together, the overall resistance landscape is characterized by diverse genetic backgrounds and multiple independent acquisition events rather than clonal expansion of a single multidrug-resistant strain. The dendrogram topology further supported these observations, with clear hierarchical separation between major resistance gene groups, reflecting distinct evolutionary and epidemiological trajectories of antimicrobial resistance in the studied population.

### 3.4. Correlations Between Resistance Genotype and Phenotype

**Figure 5:**
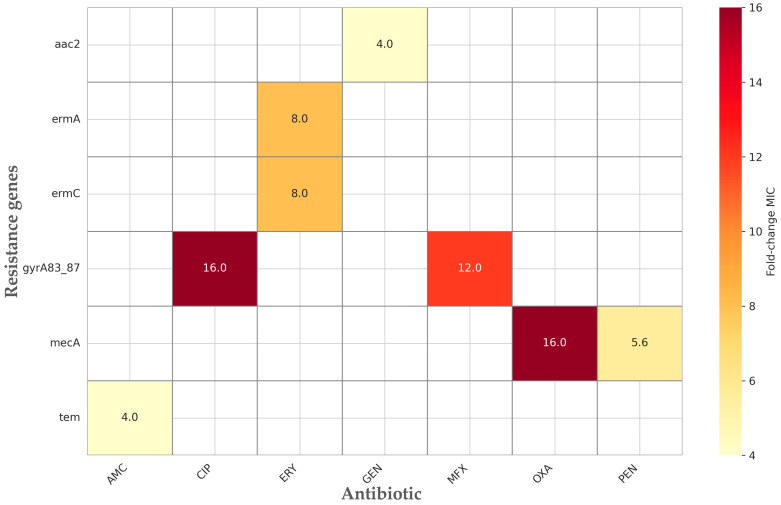
Significant correlations (*p* < 0.05) between phenotype and genotype: fold-change of MIC values.

Phenotypic susceptibility testing revealed distinct resistance patterns across the three major bacterial groups—Gram-negative bacilli, staphylococci, and streptococci (Supplementary Table S2). Gram-negative isolates showed the highest MIC values for β-lactams, with ampicillin and piperacillin exhibiting MIC90 values of 32 µg/mL and 128 µg/mL, respectively, indicating widespread resistance within *Enterobacteriaceae*. Third- and fourth-generation cephalosporins (cefotaxime, ceftazidime, and cefepime) displayed broad MIC ranges extending up to 64 µg/mL, consistent with the low but detectable presence of ESBL genes (*tem*, *shv*, and *ctx-M*). Carbapenems remained largely active, although a subset of isolates showed elevated MICs (up to 16 µg/mL for imipenem and meropenem), in line with the sporadic detection of carbapenemase determinants such as *oxa-48* and *kpc* (Figure 5, Table 2; Supplementary Table S1).

Among staphylococci, oxacillin resistance was pronounced, with an MIC50 and MIC90 of 4 µg/mL, consistent with the high prevalence of mecA. Fluoroquinolone resistance was also substantial: ciprofloxacin and moxifloxacin MIC values reached 8 µg/mL and 4 µg/mL, respectively, in *mecA*-positive and *gyrA83*/*87*-positive isolates. Aminoglycoside resistance was variable, with gentamicin MICs ranging from 0 to 16 µg/mL, reflecting the heterogeneous distribution of *aac2* and *aacA4*. Macrolide and lincosamide resistance were frequent, with erythromycin MIC90 of 8 µg/mL and clindamycin MIC90 of 8 µg/mL, correlating strongly with *ermA* and *ermC*.

In streptococci, β-lactams remained largely effective (ampicillin MIC50 0.25 µg/mL), although some isolates exhibited elevated MICs up to 16 µg/mL. Macrolide resistance was notable, with erythromycin MICs reaching 8 µg/mL, concordant with the presence of erm genes. Moxifloxacin activity remained high in most streptococcal isolates (MIC50 0.12 µg/mL), except in those harboring fluoroquinolone resistance mutations.

A direct statistical comparison of genotype–phenotype profiles (Supplementary Table S1) confirmed several highly significant associations. *mecA*-positive isolates exhibited a 16-fold increase in oxacillin MIC (median 4.00 vs. 0.25 µg/mL, *p* < 0.0001) and a 5.6-fold increase in penicillin MIC. Fluoroquinolone resistance mutations (*gyrA83*/*87*) were associated with markedly elevated MICs for moxifloxacin (12-fold increase) and ciprofloxacin (16-fold increase; *p* = 0.0004). Aminoglycoside resistance was reflected in a 4-fold increase in gentamicin MIC among *aac2*-positive isolates. For macrolides, *ermA*- and *ermC*-positive strains displayed an 8-fold elevation in erythromycin MICs (*p* < 0.05) (Figure 5, Table 2; Supplementary Table S2).

Although *tem*-positive isolates represented a small proportion of Gram-negative bacilli, they displayed significantly higher MICs for amoxicillin/clavulanic acid (4-fold increase, *p* = 0.0248), consistent with ESBL-mediated β-lactam resistance. Conversely, the *sul1* gene was absent in one phenotypically resistant Moraxella catarrhalis isolate, illustrating that certain phenotypic resistances may occur via mechanisms not covered by the molecular panel.

Logistic regression analysis (Table 2) demonstrated strong and highly significant associations between key resistance genes and their corresponding phenotypic resistance profiles. The presence of *mecA* in staphylococci was the most powerful predictor of oxacillin resistance, with an odds ratio (OR) of 159.4 (95% CI 27.3–929.7, *p* < 0.0001). Mutations in the *gyrA* quinolone-resistance determining region (codons 83/87) were significantly associated with resistance to ciprofloxacin (OR 7.34, 95% CI 2.07–26.1, *p* = 0.0016) and moxifloxacin (OR 59.4, 95% CI 3.35–1054.2, *p* < 0.0001). The aminoglycoside-modifying enzyme gene *aac2* was also strongly associated with gentamicin resistance (OR 5.63, 95% CI 1.81–17.5, *p* = 0.003). Among β-lactamase genes, tem showed a marginal association with ampicillin resistance (OR 15.9, 95% CI 0.69–365.2, *p* = 0.044), although the confidence interval was wide due to the small number of positive isolates. No statistically significant correlations were identified for shv or *ctx-M* with resistance to penicillin or cephalosporins (*p* > 0.2), nor for *qnrA*/*B*/*S* with resistance to fluoroquinolones.

These findings confirm a strong concordance between the presence of major resistance genes and elevated MIC values, particularly for β-lactams (*mecA* and *tem*), fluoroquinolones (*gyrA83*/*87*), macrolides (*ermA* and *ermC*), and aminoglycosides (*aac2*). However, a minority of discrepancies—mainly in Gram-negative species—underscore the limitations of targeted molecular panels, which may not detect all resistance mechanisms contributing to the observed phenotype.

## 4. Discussion

The results of the study demonstrate a mixed Gram-positive and Gram-negative profile, with a clear predominance of Gram-positive cocci, which has direct implications for empirical therapeutic strategies and highlights the importance of continuous regional surveillance.

*Staphylococcus aureus*, the most frequently isolated pathogen in bacterial keratitis, which presents a growing problem of methicillin resistance (MRSA), was also the top pathogen in our study. Two separate 20-year reviews from the United States have documented an increase in methicillin-resistant *S. aureus* keratitis from 1993 to 2015. This evolution is particularly concerning, given that MRSA is frequently associated with resistance to multiple classes of antibiotics, severely limiting available therapeutic options.

The detection of *Pseudomonas aeruginosa* remains clinically significant given its aggressive course and well-documented link to contact lens-associated keratitis in developed countries. *Pseudomonas aeruginosa*, one of the most virulent pathogens in contact lens-associated keratitis, presents a susceptibility to ciprofloxacin or moxifloxacin of approximately 80% worldwide for corneal isolates. However, recent studies have highlighted the presence of large proportions of antimicrobial-resistant bacteria in ocular infections, particularly in the United States, China, and India. An alarming example is the marked increase in *P. aeruginosa* resistance to moxifloxacin observed in South India between 2007 and 2009, from 19% to 52% (*p* = 0.024). This alarming trend underscores the rapidity with which antimicrobial resistance can evolve in the context of selective pressure exerted by the extensive use of antibiotics [16]. As in Romania the number of contact-lenses related keratitis is low, we identified only six strains of *Pseudomonas aeruginosa* which presented resistance to amynoglicosides and carbapenems.

The observed association between antibiotic resistance genes and pathogens demonstrate a dual epidemiological pattern: Gram-positive cocci dominate in prevalence and carry high rates of methicillin and macrolide resistance, while Gram-negative bacilli, though less frequent, exhibit concerning β-lactamase and carbapenemase-mediated resistance. This genetic landscape underscores the need for continuous surveillance and tailored empirical therapy that accounts for both the high frequency of resistant staphylococci and the potential emergence of multidrug-resistant Gram-negative pathogens in keratitis.

When comparing our data with the results reported by André et al. (2024) [12], clear similarities and divergences emerge in the genetic landscape of antimicrobial resistance among Gram-positive bacteria causing keratitis. In our study, the *mecA* gene was the most prevalent resistance determinant, detected in over 60% of staphylococcal isolates, underscoring a significant burden of methicillin resistance. This rate is way higher than that observed in the multicenter genomic study, where *mecA* was primarily confined to specific epidemic MRSA lineages such as ST5, ST8, and ST239, but not uniformly distributed across coagulase-negative staphylococci. This suggests that in our area, methicillin resistance has disseminated more widely among both *S. aureus* and coagulase-negative strains, as more than 10% are carriers of MRSA, especially in the nasal airways [17]. These can be related to finding that some staphylococcal sequence types are associated with hospital-acquired MRSA lineages that have shown particular tropism for ocular surfaces, as demonstrated by their ability to cause severe microbial keratitis with poor visual outcomes [18].

Finally, our co-occurrence analysis revealed significant associations between mecA and other determinants such as *aac2* and *ermA*/*B*/*C* genes, highlighting the multidrug-resistant potential of staphylococcal keratitis strains. The genomic work by André et al. [12] corroborates this tendency but frames it within clonal expansion of high-risk MRSA lineages. The difference may lie in our broader detection across both *S. aureus* and coagulase-negative staphylococci, suggesting a more diffuse horizontal gene transfer environment at the local level.

Glycopeptide resistance (*vanA*, *vanB*) was not present in our study, which is consistent with other studies that have reported no evidence of vancomycin resistance among keratitis-associated Gram-positive isolates [12].

Macrolide resistance genes (*ermA*, *ermB*, *ermC*) were also common in our isolates, being identified in approximately one third of Gram-positive cocci. André et al. similarly reported widespread erm gene carriage, with *ermC* predominating in coagulase-negative staphylococci [12]. Our finding of concurrent *ermA* and *ermC* in *S. aureus* indicates a broader accumulation of macrolide resistance determinants, potentially linked to local prescribing practices and mobile element exchange.

Fluoroquinolone resistance was due to *gyrA83*/*87* mutations and qnrA/B/S, most notably in *S. aureus* and *Moraxella catarrhalis*, higher than André et al.’s study [12], suggesting stronger selective pressure in our setting. This is consistent with regional reports of extensive fluoroquinolone use in ophthalmology [19].

In Gram negatives, half of the isolates of *Citrobacter freundii* and a quarter of *Klebsiella pneumoniae* isolates harbored the *gyrA83*/*87* gene mutations. This profile is consistent with published data showing that in *Enterobacteriaceae* fluoroquinolone resistance is mostly due to mutations in the QRDR region of *gyrA* [20]. In *Citrobacter freundii*, gyrA mutations associated with resistance have been documented since the 90s.

Aminoglycoside resistance was driven largely by aminoglycoside-modifying enzyme genes: *aac2* in 17 isolates and *aacA4* in 9 isolates, concentrated in coagulase-negative staphylococci, with *aacA4* also detected in three isolates of *Klebsiella pneumoniae* and two isolates of *Citrobacter freundii*. This pattern mirrors ocular reports in which CoNS frequently carry resistance determinants that confer high-level gentamicin/tobramycin resistance [21], and in *Klebsiella pneumoniae* the literature shows that resistance by *aacA4* is prevalent [22].

Notably, our co-occurrence analysis found *aacA4* to correlate with *ctx-M* (r = 0.750), consistent with widely reported linkage of *ctx-M* ESBLs on class-1 integrons, a genetic arrangement that facilitates co-selection under either β-lactam or fluoroquinolone pressure [22].

The molecular characterization of ESBL genes in keratitis isolates analyzed three genes: *tem*, *shv*, and *ctx-M*. The tem gene was detected in 1.1% of isolates. Importantly, tem genes in ocular pathogens are often located on conjugative plasmids, enabling horizontal gene transfer and dissemination of resistance across bacterial species [23]. The *shv* gene, although identified at a lower prevalence of 0.6%, had distribution in our isolates consistent with global surveillance reports that suggest *shv*-mediated resistance remains relatively uncommon in ocular infections [24]. The *ctx-M* gene represented the most clinically significant ESBL determinant in our study with 2.94%, reflecting its worldwide prominence [25]. Notably, *ctx-M* showed a strong correlation with the *oxa-48* gene (correlation coefficient = 0.750, *p* < 0.05), suggesting co-location on the same plasmid harboring multiple resistance determinants.

The *oxa-48* gene was detected in 3.67% of isolates, a prevalence consistent with the global dissemination of the *oxa-48* plasmid family, which has become increasingly widespread across clinical settings [26]. In contrast, the *oxa-23* and *oxa-24*/*40* genes were observed at very low frequencies (0.0% and 0.73%, respectively), suggesting that these carbapenemase determinants are not yet major contributors to resistance in keratitis isolates. This distribution pattern aligns with recent observations which emphasized that while carbapenemase genes are present in ocular pathogens, their prevalence remains limited compared to other resistance determinants, reflecting the broader trends of antimicrobial resistance and biofilm-associated persistence in the ocular microbiome [27].

Sulfonamide resistance determinant *sul1* was detected only in a few *E. coli* isolates, indicating limited circulation of sulfonamide resistance within ocular *Enterobacteriaceae* locally. This pattern mirrors ocular genomics reports in which *sul1*/*sul2* have been detected only in a subset of ocular *E. coli* strains rather than being ubiquitous, suggesting focal rather than generalized dissemination in eye infections [28].

Phenotypic susceptibility patterns were largely consistent with the distribution of resistance genes. The *mecA* gene showed excellent concordance with β-lactam resistance, as expected [29], producing a 16-fold increase in oxacillin MIC and a significant rise in penicillin MIC (*p* < 0.0001). Fluoroquinolone resistance mutations (*gyrA83*/*87*) were associated [30] with marked increases in ciprofloxacin and moxifloxacin MICs (up to 12–16-fold), while *aac2* correlated with a 4-fold elevation in gentamicin MICs. Macrolide resistance genes (*ermA* and *ermC*) produced an 8-fold increase in erythromycin MICs [31]. Although infrequent, *tem* was significantly associated with higher amoxicillin/clavulanic acid MICs (4-fold increase).

A few discrepancies were noted. Some Moraxella catarrhalis isolates carried *erm* genes, but macrolides were not phenotypically tested for Gram-negative species. Conversely, one Moraxella isolate showed sulfamethoxazole-trimethoprim resistance despite lacking *sul1* gene. Corynebacterium striatum could not be phenotypically assessed due to AST panel limitations.

Overall, the genotype–phenotype agreement was high for major resistance determinants, with only isolated mismatches reflecting mechanisms not captured by the molecular panel.

Rapid molecular diagnostics are increasingly recognized as a key component of antimicrobial stewardship and precision therapy. For example, the detection of resistance genes by PCR panels has been shown to reduce time to directed therapy and improve clinical outcomes. In one study the median time to active antibiotic therapy was reduced from ~50 h to ~24 h (adjusted OR for 30-day mortality 0.37) [32].

Implementation of rapid molecular diagnostics requires integration with diagnostic stewardship, clinical interpretation of results, and coordination with microbiology and infectious disease teams [33]. In the context of our study, the ability to detect *mecA, gyrA83/87, ermA/ermC* and *aac2* within hours could permit earlier optimization of empiric therapy, reduce use of broad-spectrum agents, and potentially contribute to reduced resistance selection pressure [34].

The emergence of carbapenemase genes, though at low frequency, represents a concerning trend that requires continued surveillance. The co-occurrence of multiple resistance determinants on the same mobile genetic elements suggests that selective pressure from any single antibiotic class may co-select for resistance to multiple drug classes [35].

The molecular characterization of resistance genes has direct implications for therapeutic decision-making in keratitis management. The high prevalence of *mecA* (60.2%) suggests that empirical therapy with beta-lactam antibiotics is likely to be ineffective in the majority of staphylococcal keratitis cases. Alternative agents such as vancomycin, linezolid, or newer anti-MRSA agents should be considered for severe infections.

The correlation between carbapenemase and extended-spectrum beta-lactamase genes indicates that multidrug-resistant isolates are rather common, limiting therapeutic options. This finding supports the implementation of combination therapy approaches and the development of novel antimicrobial agents with activity against resistant pathogens.

However, phenotypic resistance with absent resistant genes may be explained by incomplete expression, transcriptional silencing, or regulation by environmental conditions. In addition, some resistance determinants require specific promoter sequences, regulatory mutations, or co-factors to produce a resistant phenotype, which are not captured by targeted PCR panels.

Phenotypic resistance can occur without detectable resistance genes, due to mechanisms like chromosomal mutations (outside resistance-associated regions), increased efflux pump activity, loss of porins, biofilm formation, or adaptation to the ocular environment. These are especially relevant in Gram-negative bacteria and are not fully covered by standard molecular panels.

Testing for resistance genes only occasionally adds limited value or risks misinterpretation in bacterial keratitis, and this occurs primarily in well-defined scenarios rather than routinely. In our experience and in the context of published data, discordance between genotypic findings and clinical or phenotypic behavior represents a minority of cases, particularly when high-impact resistance determinants (e.g., *mecA*, quinolones resistance mutations, or major ESBLs) are detected.

Limited added value may arise when resistance genes are present but not phenotypically expressed, such as silent or low-level expression of β-lactamase or macrolide resistance genes, or when the detected determinant does not significantly affect the topical antibiotic concentrations achieved on the ocular surface. Nevertheless, misleading decisions may occur if molecular results are interpreted in isolation, without confirmation by phenotypic susceptibility testing or consideration of clinical evolution.

### Limitations

This study has several limitations that should be acknowledged. Whole-genome sequencing (WGS) was not performed. While the multiplex PCR-based approach provided rapid detection of key resistance determinants, it offers only partial insight into the broader resistome, virulence factors, and genomic context of resistance gene dissemination. The single-center design and the regional patient population limit the generalizability of these findings. The microbial spectrum and resistance gene prevalence observed in this study may not fully reflect those in other geographic regions or healthcare settings with different antibiotic use patterns and environmental exposures. Cost considerations must also be taken into account. Molecular diagnostic platforms such as the Unyvero system, while providing rapid and accurate results, remain relatively expensive and may not be feasible for routine use in all ophthalmology or microbiology laboratories, particularly in resource-limited settings.

Key limitations include the single-center design, modest sample size, absence of whole-genome sequencing, high cost of method, and panel scope (gene detection without QRDR sequencing). Although molecular methods provide speed and high sensitivity, discrepancies may arise between the detection of resistance genes and the actual phenotypic expression of resistance. Moreover, correlations observed between bacterial species and specific resistance genes can be misleading, since some genes may originate from bacterial species not included among the targets of multiplex PCR panels.

Therefore, rapid molecular diagnostics should not replace but rather complement phenotypic susceptibility testing, and should be embedded within institutional algorithms and stewardship programs.

## 5. Conclusions

Our study identified Gram-positive bacteria as the main pathogens involved in bacterial keratitis, and a smaller but clinically relevant contribution from Gram-negative bacilli. Co-occurrence patterns suggest plasmid-borne linkages that may drive co-selection under routine ophthalmic prescribing pressures.

The great advantage of this genotypic method is that it requires a short time from sample collection to bacteria identification and drug-resistance assessment. Thus, there is very little delay from diagnosis to targeted treatment and there is no need for empirical treatment. Multicenter studies with large numbers of samples and whole-genome sequencing could represent the cornerstone for guidelines in targeted antibiotherapy not only in ophthalmology, but in any other specialty.

Clinically, this method is fast, precise, reduces the burden of empirical antibiotherapy, and improves patient outcomes, but the cost is high, prohibitive for many centers. Therefore, the chances are high for it to remain a research tool rather than become a standard method, at least in the near future.

The present study provides essential regional data on the genotypic resistance landscape in bacterial keratitis and highlights the need for ongoing surveillance integrating molecular and phenotypic approaches. In the future, these molecular studies can be extended and compared with other patient populations within other regions of Romania.

## Figures and Tables

**Table 1:** Antibiotic resistance genes identified in clinical isolates.

	Resistance Markers																				
Species	*tem*	*shv*	*ctx-M*	*mecA*	*aac2*	*aacA4*	*gyrA83/87*	*qnrA/B/S*	*kpc*	*ndm*	*oxa-23*	*oxa-24/40*	*oxa-48*	*imp*	*vim*	*sul1*	*ermA*	*ermB*	*ermC*	*vanA*	*vanB*
*Citrobacter freundi*	0	0	0	0	0	0	2	0	0	0	0	0	0	0	0	0	0	0	0	0	0
Coagulase-negative staphylococci	0	0	2	21	17	0	2	5	0	0	0	0	2	0	0	0	2	2	4	0	0
*Corynebacterium striatum*	0	0	0	0	0	0	1	1	0	0	0	0	0	0	0	0	0	1	0	0	0
*Escherichia coli*	1	0	0	0	0	3	0	4	0	0	0	0	0	0	0	2	0	0	0	0	0
*Klebsiella pneumoniae*	1	0	2	0	0	4	2	6	1	0	0	0	2	0	0	0	0	0	0	0	0
*Moraxella catharalis*	0	0	0	0	0	0	3	3	0	0	0	0	0	0	0	0	2	0	2	0	0
*Proteus mirabilis*	1	1	0	0	0	1	1	0	0	0	1	1	0	0	0	0	0	0	0	0	0
*Pseudomonas aeruginosa*	0	0	0	0	0	2	4	0	0	0	0	0	1	0	0	0	0	0	0	0	0
*Staphylococcus aureus*	0	0	0	34	0	0	8	0	0	0	0	0	0	0	0	0	15	2	8	0	0
*Streptococcus pneumoniae*	2	0	0	0	0	0	1	0	0	0	0	0	0	0	0	0	0	1	0	0	0
*Streptococcus* spp.	0	0	0	0	0	0	3	0	0	0	0	0	0	0	0	0	2	1	1	0	0
Total	5	1	4	55	17	10	27	19	1	0	1	1	5	0	0	2	21	7	15	0	0

**Table 2:** Logistic regression analysis of the association between resistance genes and phenotypic antimicrobial resistance.

Gene	Antibiotic (Phenotype)	OR (95% CI)	*p*-Value
*mecA*	Oxacillin R (staphylococci)	159.4 (27.3–929.7)	<0.0001
*gyrA83/87*	Ciprofloxacin R	7.34 (2.07–26.1)	0.0016
*gyrA83/87*	Moxifloxacin R	59.4 (3.35–1054.2)	<0.0001
*aac2*	Gentamicin R	5.63 (1.81–17.5)	0.0034
*tem*	Ampicillin R	15.9 (0.69–365.2)	0.044

OR: Odds ratio; R: resistant.

## Data Availability

The original contributions presented in this study are included in the article/Supplementary Materials. Further inquiries can be directed to the corresponding author.

## Supplementary Materials

The following supporting information can be downloaded at https://www.mdpi.com/article/10.3390/diagnostics16010135/s1, Table S1. Distribution of MICs (µg/mL) for key antibiotics in the bacterial groups; Table S2. Significant correlations (*p* < 0.05) between resistance genes and MICs.
