# Supplementary material for: Genotypic Resistance Analysis of Bacterial Species Involved in Infectious Keratitis
Source: Diagnostics (Basel). 2026 Jan 1;16(1):135. doi: 10.3390/diagnostics16010135 (PMC12785263; doi:10.3390/diagnostics16010135)
Supplement: Supplementary file 1 [file diagnostics-16-00135-s001.zip › diagnostics-4023446-supplementary.pdf]

Supplementary Table S1. Distribution of MICs (µg/mL) for key antibiotics in the bacterial groups

Bacteria group	Antibiotic class	Antibiotic	MIC50	MIC90	MIC Interval (Min-Max)
Gram-negatives	Beta-lactams	AMP	32.00	32.00	2.00-32.00
		AMC	3.00	16.00	2.00-32.00
		PIP	6.00	128.00	1.00-128.00
		CTX	1.00	64.00	0.00-64.00
		CAZ	2.00	64.00	1.00-64.00
		FEP	1.00	64.00	1.00-64.00
		IPM	2.00	16.00	0.25-16.00
		MEM	0.25	16.00	0.00-16.00
	Fluoroquinolones	CIP	0.25	4.00	0.00-8.00
		LEV	0.12	8.00	0.00-8.00
	Aminoglycosides	AMK	2.00	64.00	0.00-64.00
		GEN	1.00	2.00	0.00-16.00
Stafilococci	Betalactams	OXA	4.00	4.00	0.25-4.00
	Fluoroquinolones	CIP	0.50	8.00	0.12-8.00
		MFX	0.25	4.00	0.00-8.00
	Aminoglycosides	GEN	0.50	16.00	0.00-16.00
	Macrolids	ERY	8.00	8.00	0.25-8.00
	Lyncosamids	CLI	0.25	8.00	0.00-8.00
	Glycopeptides	VAN	1.00	2.00	0.00-8.00
Streptococci	Beta-lactams	AMP	0.25	16.00	0.25-16.00
		CTX	0.50	2.00	0.12-4.00
	Fluoroquinolones	MFX	0.12	1.00	0.12-4.00
	Macrolids	ERY	0.12	8.00	0.00-8.00
	Glycopeptids	VAN	0.50	1.00	0.25-2.00

MIC – Minimum Inhibitory Concentration; MIC50 – Minimum inhibitory concentration required to inhibit 50% of isolates; MIC90 – Minimum inhibitory concentration required to inhibit 90% of isolates; AMP – Ampicillin; AMC – Amoxicillin/Clavulanic Acid; PIP – Piperacillin; CTX – Cefotaxime; CAZ – Ceftazidime; FEP – Cefepime; IPM – Imipenem; MEM – Meropenem; CIP – Ciprofloxacin; LEV – Levofloxacin; AMK – Amikacin; GEN – Gentamicin; OXA – Oxacillin; MFX – Moxifloxacin; ERY – Erythromycin; CLI – Clindamycin; VAN – Vancomycin.

Supplementary Table S2. Significant correlations ($p < 0.05$) between resistance genes and MICs.

Resistance gene	Antibiotic	MIC median (gene+)	MIC median (gene-)	Fold-change	p-value
mecA	OXA	4.00	0.25	16.0x	<0.0001
mecA	PEN	0.50	0.09	5.6x	<0.0001
gyrA83/87	MFX	3.00	0.25	12.0x	<0.0001
gyrA83/87	CIP	8.00	0.50	16.0x	0.0004
aac2	GEN	2.00	0.50	4.0x	0.0009
ermA	ERY	8.00	1.00	8.0x	0.0036
tem	AMC	8.00	2.00	4.0x	0.0248
ermC	ERY	8.00	1.00	8.0x	0.0344

AMC – Amoxicillin/Clavulanic Acid; CIP – Ciprofloxacin; GEN – Gentamicin; OXA – Oxacillin; MFX – Moxifloxacin; ERY – Erythromycin.
